# Glial Cell-Mediated Neuroinflammation in Alzheimer’s Disease

**DOI:** 10.3390/ijms231810572

**Published:** 2022-09-12

**Authors:** Nour F. Al-Ghraiybah, Junwei Wang, Amer E. Alkhalifa, Andrew B. Roberts, Ruchika Raj, Euitaek Yang, Amal Kaddoumi

**Affiliations:** 1 Department of Drug Discovery and Development, Harrison College of Pharmacy, Auburn University, 720 S Donahue Dr., Auburn, AL 36849, USA; 2 Division of Pharmaceutics & Pharmacology, College of Pharmacy, The Ohio State University, Columbus, OH 43210, USA

**Keywords:** Alzheimer’s disease, neuroinflammation, microglia, astrocytes, diagnostic markers, therapeutics, clinical trials

## Abstract

Alzheimer’s disease (AD) is a progressive neurodegenerative disorder; it is the most common cause of dementia and has no treatment. It is characterized by two pathological hallmarks, the extracellular deposits of amyloid beta (Aβ) and the intraneuronal deposits of Neurofibrillary tangles (NFTs). Yet, those two hallmarks do not explain the full pathology seen with AD, suggesting the involvement of other mechanisms. Neuroinflammation could offer another explanation for the progression of the disease. This review provides an overview of recent advances on the role of the immune cells’ microglia and astrocytes in neuroinflammation. In AD, microglia and astrocytes become reactive by several mechanisms leading to the release of proinflammatory cytokines that cause further neuronal damage. We then provide updates on neuroinflammation diagnostic markers and investigational therapeutics currently in clinical trials to target neuroinflammation.

## 1. Introduction

Alzheimer’s disease (AD) is a progressive neurodegenerative disorder characterized by cognitive dysfunction, memory impairment, and motor abnormalities, which could ultimately affect speech, behavior, and visuospatial orientation [1,2]. AD is the most common cause of dementia, accounting for 60 to 80% of the cases. It is characterized by two key pathological hallmarks, namely the aggregation of extracellular amyloid-β peptides (Aβ) and the build-up of aggregated neurofibrillary tangles (NFTs) throughout the brain [3,4], which result in neuronal atrophy and synaptic loss leading to neurodegeneration [5].

Aging is the strongest risk factor for AD; the prevalence is expected at 10% for individuals over 65 and 40% for those over 80 years old [6,7]. Moreover, as the world’s population ages, the frequency of AD increases, making AD a serious world healthcare challenge [8]. There are more than 55 million AD cases worldwide, projected to triple to 152 million in 2050 [9,10]. Economically, AD is one of the major public health problems, with a projected cost of USD 1.1 trillion by 2050 [11]. Accordingly, this rise in personal and financial costs suggests the necessity of efficient preclinical diagnosis and therapeutic management to prevent or halt the disease progression before the symptomatic onset [12].

At present, there is no treatment for AD. Current medications such as the cholinesterase inhibitors donepezil, galantamine, and rivastigmine, and the NMDA antagonist memantine provide symptomatic relief such as improving memory and ability to perform daily functions without curing the disease [13,14]; and the recently approved immunotherapy aducanumab, which is the only disease-modifying medication to treat AD by targeting Aβ, continues to be under trials to determine and confirm its effectiveness over time to improve memory and to hold the progression of cognitive decline [15]. While antipsychotic medications often treat secondary symptoms, such as agitation, depression, and sleep disorders [16], none of these medications halt or stop the progressive neuronal loss that leads to AD [17].

Despite decades of research, the exact etiology of AD is unknown [18,19]. However, several investigators believe that environmental and genetic risk factors play a role in AD manifestation by initiating a pathophysiologic cascade that, over decades, leads to AD pathology and dementia [3,20]. Extensive research has shown that early-onset AD (EOAD) is usually an autosomal dominant inherited disorder that represents about 1–2% of all cases [21] and involves mutations in the gene’s amyloid precursor protein (APP), presenilin 1 (PSEN1), or PSEN2. However, the majority of the cases of the sporadic type, which represent more than 95% of AD cases [21,22,23,24], have been linked to comorbidities such as insulin resistance, diabetes, hypertension, cerebrovascular and cardiovascular diseases, epilepsy, poor diet, head injury, and stress among others [25,26].

While the extracellular deposits of Aβ and the intraneuronal deposits of NFTs are the major pathological characteristics of AD [4], accumulating evidence supports that Aβ and NFTs cascades alone cannot elucidate the pathogenesis of AD, which signifies the involvement of other mechanisms in the AD pathological process [4]. Accordingly, a third core pathology in AD has emerged, supported by extensive research demonstrating that AD patients have a sustained inflammatory response in the brain suggesting the significant role of neuroinflammation in AD [27,28,29].

## 2. Neuroinflammation in Alzheimer’s Disease

Neuroinflammation plays a critical role in AD onset, pathophysiology, and progression [30,31,32,33]. The term “neuroinflammation” indicates the presence of an inflammatory response in the central nervous system (CNS) with the accumulation of glial cells, specifically the astrocytes and microglia, in response to an injury [31,34]. In the early stage of AD, brain immune cells play a neuroprotective role [30]. However, as the disease progresses, glial cells become activated, and the production of pro-inflammatory cytokines associated with oxidative stress increases, ultimately leading to neuroinflammation and neurotoxicity [31], which could further exacerbate Aβ and tau pathologies by various mechanisms.

### Factors Affecting Neuroinflammation in Alzheimer’s Disease

Multiple factors drive the onset and progression of neuroinflammation in AD that can be classified under internal and external factors. Internal factors that could potentiate neuroinflammation in AD include sex, age, and genetic factors. Sex role in neuroinflammation is mainly due to changes in steroidal hormones and estrogen receptors [35]. Estrogen receptors (ERs) were first linked to AD based on research showing that females are at a higher risk of developing AD. Besides acting as sex hormone receptors, ERs have a role in lipid and carbohydrate metabolism [36]. Furthermore, ERs signaling acts as a transcription factor providing a neuroprotective effect [37]. Previous research showed that estrogens could also regulate the formation and breakdown of Aβ [38]. Estrogen agonists possess a neuroprotective effect by decreasing neuroinflammation via reducing microglial activation and proinflammatory cytokines release, which is modulated through the activation of ERα and ERβ [39]. ERs activation induces the expression of multiple neuroprotective genes, such as 3-beta-hydroxysterol delta-24-reductase (DHCR24), which ultimately works by reducing oxidative stress [40]. β-Estradiol treatment reduced cognitive impairment in mice deficient in estrogen; this reduction was mediated by the suppression of the nuclear factor-κB (NF-κB) signaling pathway [41]. Besides sex, aging [42], and genetic mutations such as triggering receptor expressed on myeloid cells 2 (TREM2) R47H mutation [43] and ApoE4 mutation [44], all could negatively affect neuroinflammation possibly due to accumulating misfolded proteins and increasing endoplasmic reticulum stress [45]. More details on the role of TREM2 in neuroinflammation are described below under the microglia section.

With regard to external factors, many studies have shown that stress is highly correlated with the exacerbation of AD through the activation of the hypothalamic-pituitary-adrenal (HPA) axis and increased cortisol level [46]. Chronic stress in 6 and 9 month old APPswe/PS1dE9 mouse models of AD increased the brain levels of Aβ_40_ and Aβ_42_ in the hippocampus, and reduced cognitive abilities, an effect alleviated by the glucocorticoid receptors blocker, mifepristone [47]. The role of stress was also confirmed in a cohort study with three mouse models, namely, the AD mouse model Tg2576 with the APPswe mutation, the AD model PS19 harboring P301S mutant human tau, and a mouse model overexpressing corticotropin-releasing factor. In Tg2576 mice, stress increased brain Aβ levels, reduced microglial activation, and worsened cognitive abilities. In the PS19 mice, stress increased tau hyperphosphorylation and aggregation. The observed effect of stress was linked to the activation of the HPA axis as mice overexpressing corticotropin-releasing factors exhibited higher hyperphosphorylated tau compared to their wild-type control mice [48].

In addition to stress, heavy metal toxicity could increase neuroinflammation by acting as catalytic agents for redox reactions, thus, increasing oxidative stress [49,50]; alternatively, heavy metals could cause protein misfolding [51,52]. Similar to neuroinflammation, oxidative stress is a critical factor in the pathogenesis and progression of AD [53,54,55,56]. The imbalance between the production of reactive oxygen (ROS) and nitrogen (RNS) species and the antioxidant defenses results in protein oxidation and lipid peroxidation [57,58]. Studies have shown oxidative stress starts early in AD and is linked to Aβ. High levels of Aβ were associated with elevated ROS and RNS in the AD hippocampus and cortex, while low levels of Aβ in the cerebellum were associated with low oxidative stress markers [57]. Indeed, oxidative stress can be both a cause and a consequence of neuroinflammation [53]. ROS and RNS at normal physiological levels are vital in regulating numerous physiological functions. However, in a chronic oxidative stress state, these reactive species tend to be harmful by oxidizing lipids and proteins and can damage DNA [59]. Subsequently, ROS and RNS mediate signaling pathways that result in the activation of astrocytes and microglia [60]. Moreover, elevated levels of ROS and RNS are linked to high secretions of proinflammatory cytokines such as interleukin-6 (IL-6), interleukin 1β (IL-1β), and tumor necrosis factor-α (TNFα), interferons, and chemokines [59,61,62]. Such release of proinflammatory cytokines triggers the microglia to produce plentiful amounts of ROS and RNS, which lead to NF-κB activation [63,64], and, subsequently, the overexpression of nitric oxide (NO), specifically NOX2, inducible nitric oxide synthase (iNOS), cyclooxygenase-2 (COX-2), and cytosolic phospholipase A2 [62].

Additional external factors that could induce neuroinflammation include metabolic disorders such as obesity and diabetes [65] and the western diet [66,67]. The chronic consumption of the western diet could cause obesity and gut microbiota dyshomeostasis [68,69]. Long-term obesity and high-fat diet were associated with depression, anxiety, and increased brain inflammatory markers [70]. In addition, the western diet has been linked to microbiota alterations and endotoxins production, which have been linked to the blood–brain barrier (BBB) disruption resulting in BBB leakage of blood toxins to the brain and neuroinflammation [71]. Several studies have identified the link between the brain and the gut microbiome, known as the microbiota-gut–brain (MGB) axis [72,73]. This link could affect neuropathological diseases positively or negatively [72]. The MGB axis is bidirectional, where the gut microbiome produces short fatty acids that regulate the integrity and function of the BBB and the gut. The brain affects the gut via the vagus nerve and hypothalamus–pituitary axis [73]. The gut microbes activate the CCAAT/enhancer binding protein β/asparagine endopeptidase (C/EBPβ/AEP) pathway and elevate neuroinflammation and cognitive impairment [74]. A comparison of gut microbiota between the AD mouse model 5XFAD and wild-type mice demonstrated an alteration of gut microbiota composition that led to the accumulation of phenylalanine and isoleucine, which stimulate pro-inflammatory reactions [75]. Some microbial metabolites, such as the microbiota-derived short-chain fatty acids (SCFA), promoted Aβ deposition and increased microglia activation in APP/PS1 mice [76]. The microbiota-induced neuroinflammation could be reversible. For example, Lin and colleagues reported that treatment of the AD mouse model 3xTg with hydrogen-rich water could alter the gut microbiota, an effect associated with less cognitive impairment, fewer Aβ deposits, less phosphorylated tau, and reduced microgliosis [77]. In humans, patients with AD have shown less microbiota diversity and richness compared to age and gender-matched cognitively normal individuals [78]. Indeed, more research is required to clarify the role of microbiota in AD and the potential of targeting gut microbiota as a therapeutic approach to treat AD.

While the literature contains a large body of research on neuroinflammation, here, we provide a concise mechanistic overview of glial cells’ role in neuroinflammation associated with AD, available diagnostic tools, and therapeutics that target neuroinflammation currently in clinical trials.

## 3. Glial Cells Role in Neuroinflammation

Neuroinflammation is usually linked to the chronic activation of microglia and astrocytes. While their contribution to AD is established and reported, further research focusing on glial cells’ role in health and disease is necessary to identify new targets for the development of diagnostic tools and therapeutic strategies against AD.

### 3.1. Microglia

**Figure 1:**
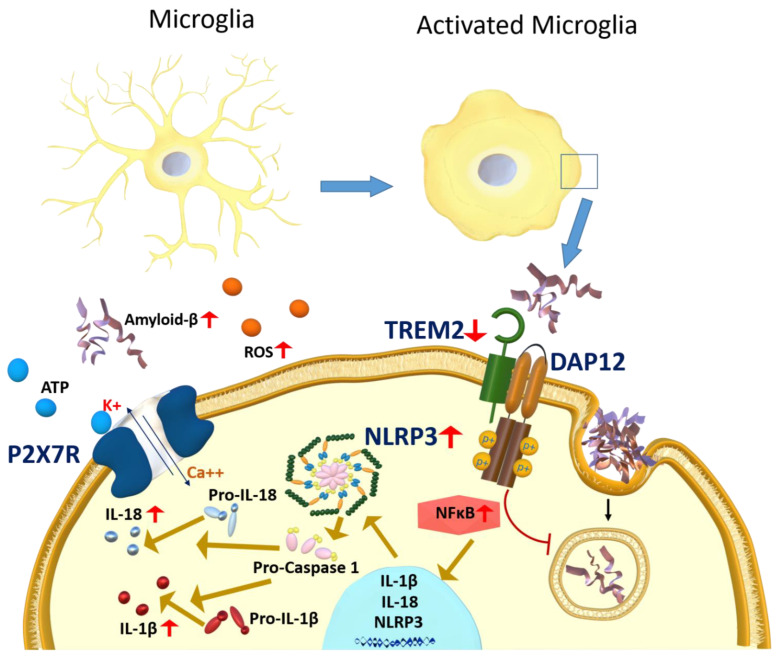
Microglia activation in AD. Neuroinflammation in AD is associated with microglia activation that is mediated by increased Aβ, ROS, and ATP, which could activate P2X_7_R and downregulate TREM2. P2X7R activation would increase calcium influx, which leads to microglia activation and the release of inflammatory cytokines. In AD, reduced levels of TREM2 would impair Aβ phagocytosis by the microglia and thus increase Aβ brain parenchymal burden. Microglia activation is also associated with NF-κB and NLRP3 inflammasomes activation, which would activate caspase-1 and the secretion of proinflammatory cytokines IL-1β and IL-18.

Microglial cells are the innate immune cells in the CNS, which first appear as colonies in the embryonic brain, and as the brain develops, microglia migrate all over the CNS [79]. Microglial cells are resident phagocytes that play critical roles in CNS maintenance, as first-line pathogen defense, and injury response [80]. Thus, under normal physiologic conditions, microglia play a role in immune surveillance. However, microglia become activated under pathological conditions such as neurodegenerative diseases, stroke, and tumor invasion [81]. In the early stages of AD, activated microglia have a positive role in clearing Aβ by phagocytosis. Nevertheless, after prolonged exposure, their efficiency to clear Aβ is reduced and starts to negatively affect the brain leading to Aβ accumulation, which subsequently forms extracellular plaques that continuously stimulate microglial activation [82]. The overactivation of microglia adopts them to the reactive states [83], characterized by significant phenotypic and morphology changes accompanied by elevated expression of the pro-inflammatory cytokines TNFα, IL-6, IL-1β, and NO [83,84]. This aggressive status of microglia creates a chronic neuroinflammatory environment and exacerbates neuronal and synaptic loss [85,86,87]. In this section, we briefly review selected targets involved in microglial activation, which are also summarized in Figure 1.

#### 3.1.1. P2X_7_R Role in Microglia Activation

Microglia activation is mediated by several mechanisms, including the purinergic receptor P2X_7_ receptor (P2X_7_R) (Figure 1). Recently, the role of P2X_7_R in neurodegenerative diseases, including AD, has been thoroughly reviewed [88,89,90]. P2X_7_R role in reactive microglia is evident from studies utilizing P2X_7_R knock-out mice where the stereotaxic injection of Aβ (2.2 μM in 1 μL of artificial cerebrospinal fluid (CSF)) into the dorsal hippocampus failed to activate microglial cells [91]. P2X receptors are plasma membrane ion channels activated via ATP binding and are upregulated in AD. Of this family, P2X_7_R is more sensitive to ROS/NOS, NFκB, and NACHT, LRR, and PYD domains-containing protein 3 (NLRP3) inflammasome activation, as well as ATP [88]. In AD, Aβ triggers the immune cells and activates them to release ATP. ATP release activates P2X_7_R, which further activates microglia to release inflammatory cytokines that trigger neuroinflammation [92,93]. P2X_7_R is overexpressed in the microglia surrounding senile plaques in the hAPP transgenic mouse model J20 expressing EGFP downstream of the P2X_7_R promoter (^P2X7R^EGFP/J20). While the overexpression of P2X_7_R allows increased microglial migration to Aβ plaques, the phagocytotic capacity of the reactive microglia is reduced. This effect was blocked by the selective P2X_7_R antagonist GSK 1482160A, which increased the migration and phagocytic capacity of microglial cells and reduced neuroinflammation [94]. P2X_7_R inhibition is proposed as a therapeutic approach to reduce neuroinflammation in AD [95].

#### 3.1.2. RAGE Role in Microglia Activation

**Figure 2:**
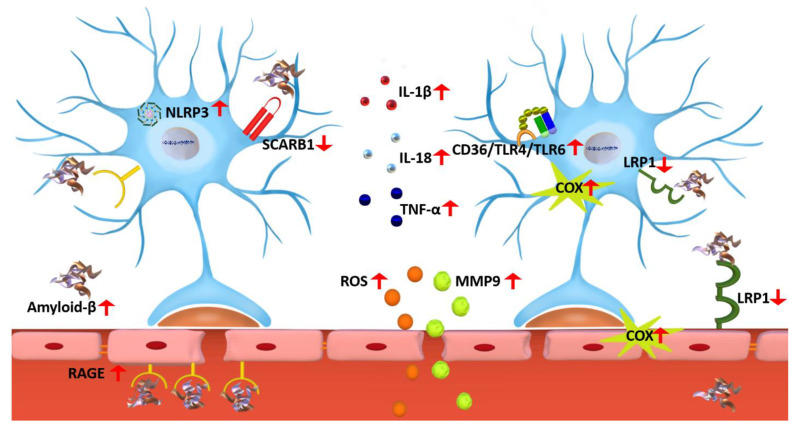
A representative scheme demonstrates astrocytes’ interaction with the BBB and the neuroinflammation effect mediated by astrocyte activation on the BBB function. In AD, reduced expression of astrocytic SCARB1 and LRP1 would decrease the clearance of Aβ. Reactive astrocytes produce a wide range of cytokines and other inflammatory markers such as ROS, RAGE, COX, and MMP9 due to NLRP3 inflammasomes and NF-κB pathways activation. In addition, TLR4 and TLR6 activation through binding to CD36 would increase proinflammatory cytokines secretion leading to neuroinflammation and BBB dysfunction. In AD, the expression of endothelium RAGE and LRP1 are upregulated and downregulated, respectively, which lead to reduced LRP1-mediated clearance of Aβ, and increased RAGE-mediated Aβ influx into the brain, which would further increase the vicious cycle of brain Aβ accumulation, astrocytes activation, and BBB dysfunction. Astrocytes, and microglia, could also be activated via TLRs. TLRs are pattern recognition receptors (PRR) that recognize and bind substrates and activate the immune response [186]. TLR could be expressed on the cell surface as TLR1, 2, 4, 5, 6, and 10 or intracellular as TLR 3, 7, 8, and 9, and their expression varies among immune cells [186]. In the immune cells of human AD brains, TLR mRNA is overexpressed except for TLR2, which is not changed [186]. Aβ is a substrate for TLR; upon binding, it stimulates its overexpression [187]. Recent research showed that while Aβ_42_ did not affect the expression of TLR3, it increased its reactivity in cultured microglial cells [188]. TLR4 antagonists could reduce neuroinflammation in AD, yet they might interfere with Aβ clearance by lowering the microglial phagocytotic ability of Aβ. In contrast, TLR4 agonists might have a beneficial role in Aβ clearance but not in reducing neuroinflammation [189]. Other researchers reported that the deletion of TLR2 in APPswe/PSEN1dE9 transgenic mice caused cognitive impairment, and increased anxiety, white matter damage, and brain atrophy in an Aβ-independent pathway [190], which collectively highlights the controversy regarding the role of TLR in AD [191].

The receptor for advanced glycation end products (RAGE) is expressed on an array of immune-related and non-immune-related cells, such as the BBB-endothelium cells [96]. RAGE has been implicated in Aβ pathology, most importantly, in the access of peripheral Aβ through the BBB to the brain (Figure 2) [97]. In AD, endothelium–RAGE is upregulated, suggesting its role in increased brain Aβ levels due to increased Aβ influx from the blood to the brain via RAGE [98,99]. This was shown in Tg2576 mice, where RAGE expressed on the luminal side of the endothelial cells of the BBB facilitated the transport of Aβ_40_ and Aβ_42_ transfused through the carotid artery to the mouse brain [100]. The specific role of RAGE was confirmed by infusing a RAGE-specific IgG that blocked RAGE-mediated Aβ transport across the BBB. RAGE’s role in Aβ transport across the BBB was further confirmed in homozygous RAGE knockout mice where infused Aβ could not be detected in the brain. RAGE-mediated Aβ transport to the brain was associated with increased proinflammatory cytokines, including IL-6 and TNF-α. Besides its expression in endothelial cells, RAGE is expressed in microglia. Its upregulation induced mitochondrial damage, oxidative stress, and impaired mitophagy flux in a stress model in wild-type mice [101]. The overexpression of microglial RAGE in a mouse model expressing human mutant APP further activated microglia to release more IL-1β and TNF-α compared to APP mice carrying transduction-defective microglial RAGE [102]. In a Parkinson’s disease mouse model, the silencing of RAGE reduced neuroinflammation because of NF-κB pathway suppression and COX-2 downregulation [103]. Besides Aβ, RAGE is activated by many other ligands, including the danger-associated molecular patterns (DAMPs), such as the high mobility group box 1 (HMGB1) [104]. HMGB1 is expressed within the nucleus as a mediator of transcriptional processes and in the cytoplasm, where it regulates autophagy [105]. However, upon its translocation outside the cell, HMGB1 interacts with RAGE to regulate immune processes through the upregulation of proinflammatory cytokines and chemokines [106]. RAGE and HMGB1 are upregulated in AD, so their interaction leads to the activation of several inflammatory signaling pathways, including NF-κB and cell death [96,107]. For additional information on the role of RAGE in AD, readers can refer to the review [108].

#### 3.1.3. NLRP3 Inflammasome Activation in Response to Microglia Activation

In AD, Aβ acts as a stimulus that activates NLRP3 inflammasomes. NLRP3 inflammasome consists of multiple cytosolic protein complexes [109] that, upon activation, would activate caspase-1 and the secretion of the proinflammatory cytokines IL-1β and IL-18 (Figure 1) [110]. The activation of NLRP3 inflammasomes has also been linked to tau pathology. For example, NLRP3 inflammasome loss of function mutations reduced tau pathology and prevented cognitive decline in the heterozygous THY–Tau22 transgenic mouse model [111]. The serotonin receptor antagonists and reuptake inhibitor, trazodone, reduced memory impairment and sleep disturbances in the rTg4510 mouse model of AD by reducing the neuroinflammation resulting from tauopathy mediated by reducing microglial and NLRP3 inflammasomes activation [112]. The inhibition of NLRP3 inflammasome activation by the NLRP3 inhibitor MCC950, an investigational drug inhibitor, in a streptozotocin-sporadic AD mouse model was protective against the pathological reactive microglia [113]. Currently, NLRP3 inflammasomes are evaluated as a therapeutic target for AD by regulating the neuroinflammatory response driven by NLRP3 [114].

IL-1β is released as a part of the innate immune response by NLRP3 inflammasome activation [110]. IL-1β is overexpressed in the brains of AD patients [115]. Increased levels of IL-1β have been linked to Aβ plaques, tau hyperphosphorylation, and NFTs [116]. IL-1β induction with hippocampal LPS injections caused memory impairment in rats by inducing microglial activation [117]. IL-1β blocking with IL-1β antibodies alleviated memory and cognitive dysfunction in 3xTg-AD mice by suppressing NF-κB activation and reduced tau phosphorylation by decreasing the activation of tau-related kinases cdk5/p25, GSK-3β, and p38-mitogen-activated protein kinase (p38 MAPK) [118]. Thus, IL-1β antibodies are also suggested as potential therapeutic agents for AD by targeting neuroinflammation in the late stages of the disease [119]. The role of microglia and NLRP3 inflammasomes-mediated neuroinflammation in AD and its targeting as a therapeutic approach are further reviewed [120,121,122].

#### 3.1.4. TREM2 Role in Microglia Activation

Recently, the role of TREM2 in activating the immune response and neuroinflammation has been recognized [123,124]. TREM2 is a transmembrane protein receptor of the innate immune system that is exclusively expressed by microglia in the brain [125,126]. Aβ activates TREM2 signaling [115], which is also responsible for Aβ phagocytosis (Figure 1) [127]. TREM2 p.R62C and p.R62H loss-of-function variants have increased Aβ plaque seeding and microglial clustering around the plaques leading to neuroinflammation [127]. A recent meta-analysis study described a changing role of TREM2 as AD progresses. In the early stages of AD, TREM2 knockdown reduced the secretion of cytokines and other inflammatory markers in 35 different transgenic mouse models, while its knockdown from microglia in later stages induced neuroinflammation by increasing the secretion of proinflammatory cytokines [128].

Higher levels of soluble TREM2 in the CSF were associated with lower Aβ accumulation in APP^NL-G-F^, a mouse model of AD [129]. In humans, studies utilizing positron emission tomography (PET) and CSF monitoring of TREM2 demonstrated that increased TREM2 levels were associated with reduced Aβ and tau levels [129]. Additional studies showed that TREM2 deletion is associated with increased tau pathologies and hippocampal atrophy in the presence of Aβ in the TauPS2APP mouse due to disease-associated microglia activation that is Aβ- and TREM2-dependent [130]. Furthermore, it has been shown that TREM2 regulates calcium signaling in human-induced pluripotent stem cells-derived microglia (iPSCs-derived microglia); the deletion of TREM2 was associated with increased calcium signaling due to increased intracellular calcium release from the endoplasmic reticulum stores. Increased calcium reduced the motility of the cells and, therefore, the directional chemotaxis of microglia to Aβ plaques [131]. The downregulation of TREM2 further induced cognitive dysfunction, Aβ accumulation, and neuroinflammation in APP/PS1 mice. The increase in neuroinflammation was due to the activation of the toll-like receptor 4 (TLR4) mediated MAPK signaling pathway, which increased the release of proinflammatory cytokines [132]. On the other hand, the overexpression of TREM2 in BV-2 cells increased Aβ clearance and reduced neuroinflammation by downregulating the expression of TLR2, 4, and 6 [133]. The importance of TREM2 was also demonstrated in the 5xFAD mouse model of AD, where TREM2 overexpression reduced the hippocampal neuroinflammation, while TREM2 deficiency induced forkhead box protein O3 (FoxO3a) activation and deactivated phosphoinositide 3-kinases/protein kinase B (PI3K/PKB) signaling pathway, thus, increased neuroinflammation [134]. Figure 1 summarizes the primary targets that are involved in microglial activation.

#### 3.1.5. Post-Translational Modification in Microglia Activation

Post-translational modifications of proteins have been linked to neuroinflammation and the neuropathology of AD [2,135]. Recent reports have highlighted the role of protein citrullination in several immunological and inflammatory conditions, such as rheumatoid arthritis [136]. Citrullinated proteins are characterized by the deimination of the guanidinium group of the arginine side chain, mediated by peptidyl arginine deiminases enzymes, which leads to the formation of nonstandard amino acid citrulline in the protein. In AD, Aβ citrullination is associated with microglial activation [137]. While further investigations are required to clarify the mechanism(s) of this activation, increased protein citrullination in AD could be due to the activation of peptidyl arginine deiminases enzymes and Ca^2+^ dyshomeostasis [138,139]. The post-translational modifications of Aβ have also been shown to affect its interaction with TREM2. Compared to non-modified Aβ variants, TREM2 preferentially interacts with oligomers formed by phosphorylated Aβ variants for their uptake and phagocytosis by microglia [140]. For example, the phosphorylated Aβ variants pSer8-Aβ and pSer26-Aβ showed differential deposition. pSer8-Aβ deposits primarily in the core of extracellular plaques. On the other hand, pSer26-Aβ showed limited deposition in these plaques and was not detected within microglia, suggesting pSer26-Aβ oligomers might efficiently bind to TREM2 and become internalized by the microglia through TREM2 binding [140,141].

Glutaminyl cyclase (QC) is another important enzyme for post-translational modification that converts the N-terminal glutaminyl and glutamyl into pyroglutamate (pGlu) through cyclization. The secretory (sQC) and golgi resident (gQC) isoforms of the enzyme are involved in AD pathology. During the development of AD, the expression of sQC and gQC and the production of pGlu-Aβ are increased [142]. gQC is involved in the C-C motif chemokine ligand 2 (CCL2) maturation. CCL2 is one of the critical mediators of neuroinflammation in AD [143,144]. The immune cells surrounding Aβ plaques produce high levels of inflammatory molecules, including CCL2, which attracts microglia cells to the inflammation site, thus, promoting neuroinflammation [145]. In the brains of AD patients, gQC and CCL2 expressions are upregulated compared to control brains, which were correlated with pGlu-Aβ formation [146]. The above studies demonstrate a critical role of proteins post-translational modifications in reprogramming microglia homeostasis and neuroinflammation in AD. Understanding how post-translational modifications could affect AD development and progression could provide insights into therapeutic strategies to prevent or treat neuroinflammation in AD.

### 3.2. Astrocytes

Astrocytes are specialized glial cells, found predominantly in white and grey matter in the form of fibrous and protoplasmic, respectively [147]. Astrocytes typically play a regulatory role in brain function, including the BBB function, neurogenesis, synaptogenesis, and the maintenance of neurotransmitter and fluid homeostasis [148,149,150]. Furthermore, astrocytes form specialized perivascular channels in the glymphatic system, which acts as a clearance pathway of neurotoxic wastes, including Aβ and tau [147]. In AD, astrocytes become activated and undergo a series of conformational, transcriptional, and functional changes collectively referred to as reactive astrocytes [149]. Reactive astrocytes demonstrate hypertrophic processes and overexpress glial fibrillary acidic protein (GFAP), nestin, and vimentin [149,151]. Moreover, similar to the activated microglia, activated astrocytes increase the production of a wide range of inflammatory cytokines such as IL-1β, IL-6, and TNF-α [149], thereby initiating a harmful cascade that leads to impairments in neuronal functions [152]. A recent finding obtained from CRISPRi screens with single-cell transcriptomics revealed a cocktail of inflammatory cytokines (IL-1α+TNF+C1q) that trigger at least two astrocyte reactions. These reactions oppose one another and are controlled by STAT3; one is induced by STAT3 and the release of IL-6, while the other is inhibited by STAT3 and the unleash of interferons. Thereby initiating a harmful cascade that leads to impairments in neuronal functions [153].

Astrocytes clear Aβ through various mechanisms [154]. Astrocytes are capable of Aβ uptake by using transport proteins and receptors such as low-density lipoprotein receptor-related protein (LRP1), scavenger receptor class B member 1 (SCARB1), and RAGE [155,156]. Furthermore, astrocytes mediate Aβ clearance by endosomal-lysosomal pathways that degrade Aβ by neprilysin (NEP), insulin-degrading enzyme (IDE), and matrix metalloproteinase-9 (MMP9) enzymes [157,158,159]. Besides the endosomal-lysosomal pathways, astrocytes have a role in parenchymal Aβ clearance by secreting ApoE, which enhances Aβ clearance via LRP1 [155]. Astrocytes have a significant role in reducing Aβ brain parenchymal burden. However, in AD, reactive astrocytes’ capacity to clear Aβ is reduced, leading to increased Aβ levels and Aβ plaques formation [160].

In AD, the astrocytes become more sensitive to calcium in response to ATP and glutamate than normal astrocytes [161,162], leading to elevated levels of ROS and reduced levels of astrocytic glutathione (GSH), thus reducing the cellular defense mechanism against oxidative stress [163]. GSH is the most abundant non-protein thiol [164]; it is considered the primary antioxidant defense molecule in the brain [165,166] and plays a crucial role in the maintenance of redox homeostasis in neurons [167]. The intracellular levels of GSH determine the cell response to oxidative stress, and its depletion exacerbates oxidative damage [168]. Many studies suggested that the depletion and alteration of endogenous antioxidant systems such as GSH precede oxidative stress [165,169]. The reduction in intracellular GSH is associated with TNF-α and IL-6 release, and activation of the inflammatory pathways P38 MAPK, and NF-κB, in microglia and astrocytes [170,171]. Astrocytes are the main supplier of GSH to microglia and neurons [172,173]. Reduced GSH levels in astrocytes could enhance neurodegeneration as they become inflammatory cells and neglect their neuroprotective function [174,175,176]. In addition, the AD-astrocytes have a compromised energy metabolism, and their uptake and release of glutamate may be compromised [43]. For example, Simpson et al. demonstrated a reduction in the expression of the main regulator of extracellular glutamate levels, namely excitatory amino acid transporter 2 (EAAT2), which is expressed primarily by the astrocytes, suggesting the loss of astrocytes’ function to protect neurons by clearing neurotoxic extracellular glutamate [177]. Collectively, the chronic exposure of astrocytes to elevated Aβ levels would transform astrocytes from basal to reactive state and switch from metabolic support cells for the neurons to inflammatory cells, which could lead to a breakdown between astroglia and neuronal interactions, thus neglecting their neuro-supportive role [162,178].

Besides their neuro-supportive function, astrocytes play an important role in maintaining the BBB function. The BBB is a dynamic barrier that regulates and provides a constant optimal environment for neuronal function by keeping out harmful and toxic substances from the CNS [179]. The BBB acts as a selective barrier that regulates the movement of molecules entering the brain, thus keeping red blood cells, leukocytes, neurotoxic plasma-derived components, and pathogens out of the CNS [180]. The BBB comprises endothelial cells (ECs), pericytes, basement membrane, and astrocytes [179]. ECs are connected by tight junction proteins supported by junction adhesion molecules (JAMs), thus forming a physical barrier. During pathogen attacks, the BBB properties undergo significant changes, disrupting its integrity and function and leading to inflammatory reactions and neurological disorders [181].

BBB disruption has been associated with aberrant vessel regression, brain hypoperfusion, angiogenesis, inflammatory responses, and accumulation of Aβ and tau in AD. These events cause an accumulation of neurotoxic hemoglobin and iron in the brain, increasing ROS production and oxidative stress in the brain [181]. Reactive astrocytes would affect the BBB-endothelium by generating ROS and inflammatory markers such as COX and NOS [182]. This collectively could increase pro-inflammatory mediators and MMP9 activation subsequent to NLRP3 inflammasomes and NF-κB pathways activation [183], cytoskeleton rearrangement, downregulation of tight junction proteins, and eventually BBB dysfunction [184,185]. Figure 2 summarizes the effect of neuroinflammation-mediated BBB disruption by activated astrocytes.

The activation of TLR can be initiated through binding CD36 (cluster of differentiation 36) and TAM (Tyro3, Axl, and Mer) receptors. CD36, a scavenger membrane receptor known as fatty acid translocase (FAT), has a role in fatty acid transportation and is expressed in the endothelial cells of the BBB and glial cells [192]. Under normal conditions, this receptor helps clear Aβ across the BBB and through phagocytosis by the astrocytes [193]. In AD, CD36 forms a complex with TLR4 and TLR6 that is responsible for the secretion of the proinflammatory cytokine, including IL-6, TNF-α, NO, and chemokines [194] (Figure 2), which activates inflammatory signaling in the astrocytes through the PI3K/Akt signaling pathway [192]. CD36 activation increases oxidative stress by increasing ROS and is involved in cognitive decline in Tg2576 mice [195].

TAM receptors also regulate TLRs. TAM receptors are a subgroup of tyrosine kinase receptors, consisting of three kinases: tyrosine-protein kinase receptor 3 (Tyro3), tyrosine-protein kinase receptor UFO (Axl), and MER proto-oncogene (Mertk). They are expressed in neuronal cells as well as microglia and astrocytes. In normal physiological conditions, TAM receptors contribute to synaptic plasticity, neuronal migration, and the negative regulation of inflammatory response [196,197]. In AD, TAM receptors activate the microglia to phagocyte Aβ, thus inhibiting Aβ plaques formation due to Axl activation [198]. TAM receptor activation inhibits TLR activation, which reduces the release of inflammatory cytokines and decreases neuroinflammation, as seen in Tyro3/Axl/Mer knockout mice, characterized by hyperactive immune cells and hyperresponsiveness to TLR activation. TAM receptor activation is associated with reduced inflammatory response [199].

## 4. Diagnosis and Biological Markers

One growing area of research is the use of biomarkers of neuroinflammation as a diagnostic tool for AD. Methods for neuroinflammation diagnosis are still under development and are not yet used as a definitive diagnostic tool [200]. Early diagnosis of AD could lead to better treatment. Along with the early diagnosis, biomarkers could provide an objective measure of drug efficacy and disease progression [201]. However, due to the complexity of AD pathogenesis and the commonality of neuroinflammation with other neurodegenerative diseases, it is unlikely that a neuroinflammatory biomarker(s) singly be used to diagnose AD [202]. It has been recommended that sets of multiple biomarkers besides neuroinflammation be used to predict, diagnose, and track stages of AD [202].

A vast body of work on potential biomarkers has been reported. Here, we describe some major biomarkers studied for AD diagnostics, summarized in Table 1.

### 4.1. TPSO

Currently, the translocator protein 18 kDa (TSPO) expressed by microglia surrounding Aβ plaques [203] is considered the most used target for PET imaging to identify neuroinflammation in potential AD patients [204,205]. Increased PET scan signals of TSPO have been correlated to higher microglial activation and, thus, neuroinflammation [204,206]. Hippocampal TSPO alteration from astrocytes preceded those due to microglia in TgF344-AD rats and AD patients, indicating the imaging changes based on the disease stages [207]. TSPO accumulation in the 3xTg AD mouse hippocampus started before Aβ plaques formation. The increase in TSPO varied across different hippocampal areas, where the subiculum was the earliest, and the ventral was the latest [208].

Neuroinflammation imaging utilizing TSPO as a human biomarker is being used in research; however, there are two main concerns with using TSPO as a target. The first concern is the tracers used to target TSPO. [^11^C]-PK11195 was one of the first tracers used in studies of AD patients. These studies showed contradictory results because of a lack of the tracer’s specificity and sensitivity to TSPO, causing low signal-to-noise ratios in the PET scans [204,209]. This has led to the creation of second and third-generation tracers that showed the potential for more accurate assessments of neuroinflammation [204,209]. While promising, the continued development of better tracers is necessary to optimize the use of TSPO as a target [204]. The second concern with the use of TSPO which is not exclusively found in glial cells as it has also been found in endothelial cells, and vascular smooth muscle cells [209], suggesting that changes in PET imaging signals using TSPO may be due to changes in TSPO from other sources not related to neuroinflammation [209,210].

One limitation of PET scans is that they can be expensive and time-consuming, which slows research and limits large-scale use. Therefore, there has been a rapid expansion of research on biomarkers found in CSF and blood. Many biomarkers have been found in the CSF, with some being more consistent than others.

### 4.2. YKL-40

YKL-40 or chitinase-3-like protein 1 is a carbohydrate-binding protein secreted by activated microglia [211]. It has been shown that YKL-40 increases due to neuroinflammation [201,211]. This has been supported by a study that compared healthy controls (*n* = 36) to patients with either mild cognitive impairment (MCI, *n* = 39) or AD dementia (*n* = 27), which demonstrated higher levels of YKL-40 in AD patients compared to controls in CSF samples [212]. Another study that compared cognitively unimpaired elderly (*n* = 508), MCI patients, and AD dementia patients demonstrated that CSF levels of YKL-40 were increased in the preclinical, prodromal, and dementia stages of AD [213]. A recent meta-analysis study focusing on YKL-40 concluded that YKL-40 could be used in combination with other biomarkers to help in the prognosis of MCI and the likelihood of continued progression to AD [214]. With this known potential as a biomarker, a new study was designed to analyze YKL-40 levels in the brain of AD and other neurodegenerative diseases. Results from post-mortem brain levels of YKL-40 were similar across all groups. It was concluded that while CSF levels of YKL-40 increase in AD patients, it may not reflect the changes in YKL-40 in the brain [215].

### 4.3. sTREM

Another possible biomarker is sTREM2. TREM2 can undergo proteolytic cleavage producing a soluble form that is excreted into the interstitial space and CSF [201,216]. Studies comparing CSF levels of sTREM2 in controls compared to MCI and AD patients have shown a significant increase in sTREM2 in MCI and AD patients [212,217]. These results have given hope that sTREM2 can be used as a reliable marker for microglia activation.

### 4.4. VICAM-1/ICAM-1

The next plausible biomarkers are vascular cell adhesion molecule-1 (VCAM-1) and intercellular adhesion molecule-1 (ICAM-1), which are both cell-surface glycoproteins that mediate the adhesion of leukocytes to endothelial cells and assist in the transport of leukocytes to the brain [218]. Elevated levels of these receptors in CSF could allow them to be used as valuable biomarkers [218,219]. Studies have shown that CSF levels of VCAM-1 and ICAM-1 are increased in the preclinical, prodromal, and dementia stages of AD, similar to YKL-40 [213]. CSF levels of VCAM-1 and ICAM-1 increase with neuroinflammation and have already been used as reference inflammatory markers in AD patients [219]. Plasma levels of VCAM-1 and ICAM-1 have also increased in AD patients compared to control groups [218]. VCAM-1 has more consistently been shown to be increased in plasma, but there has been contradictory evidence concerning plasma levels of ICAM-1, which necessitate additional investigation [218,220]. As VCAM-1 and ICAM-1 are markers of vascular endothelial dysfunction instead of directly related to inflammation; one study concluded that these biomarkers are independent of neuroinflammation and not directly correlated [221].

### 4.5. GFAP

Glial fibrillary acidic protein (GFAP) is an important cytoskeletal component of astrocytes released into the CSF due to neuroinflammation [201,222]. It is a promising biomarker as studies have shown increased levels of GFAP in both the CSF and plasma with neuroinflammation [223,224,225]. Currently, GFAP mechanisms and pathways of release into plasma are not fully understood, limiting its ability to be used diagnostically [223].

### 4.6. MCP-1

Another possible biomarker is monocyte chemoattractant protein-1 (MCP-1) [226]. It is a chemokine that attracts microglia and peripheral immune cells responding to inflammation and has a role in activating microglia [201]. Studies have shown increased MCP-1 in AD patients compared to controls in CSF samples [212,227]. While there is a hope that MCP-1 could be used as a blood biomarker, there have been conflicting findings on whether MCP-1 levels change in plasma is, in fact, due to neuroinflammation [228,229]. Another concern could be that MCP-1 plays a vital role in inflammation throughout the body, which makes CSF, but not blood, levels of MCP-1 more likely indicative of inflammation specific to the CNS [230].

### 4.7. S100B

Another potential biomarker is S100 calcium-binding protein B (S100B). S100B is a proinflammatory cytokine found predominately in astrocytes and has concentration-specific effects. It has a neurotrophic effect promoting growth and recovery at a nanomolar concentration but has deleterious effects at micromolar concentrations [231]. While S100B has been a suggested marker of brain damage and other CNS injury, it has also increased during inflammation [232]. Studies have shown that CSF levels of S100B increase in AD patients compared to controls [233,234]. There is also potential for S100B to act as a blood biomarker for AD. A recent meta-analysis study of 32 articles (*n* = 3204 subjects) found that blood levels of S100B were higher in AD patients than in controls [224]. However, there are claims that CSF and blood levels of S100B are inconsistent, limiting their usefulness in monitoring disease progression [156].

**Table 1:** An overview of biomarkers with increased expression due to AD-related neuroinflammation.

Biomarkers	Imaging/Biological Sample	Reference
TSPO	PET	[204,206,235,236]
YKL-40	CSF	[212,213]
sTREM2	CSF	[212,217]
VCAM-1	CSF, Blood	[213,218,237]
ICAM-1	CSF, Blood	[213,218,220]
GFAP	CSF, Blood	[223,224,225]
MCP-1	CSF, Blood	[212,227,228,229]
S100B	CSF, Blood	[156,224,233,234]

Many other neuroinflammation biomarkers have been suggested, including cannabinoid receptor type 2 (CB2R), COX-1, and COX-2, all found in microglia beside other cells; however, the most promising targets should be found almost exclusively in microglia or astrocytes [209]. For example, the colony-stimulating factor 1 Receptor (CSF1R) and the P2X_7_R are found almost exclusively in microglia as they are all essential for their survival and function [209]. These receptors have much potential as targets for neuroinflammation, but more research is necessary to confirm their application [209]. Reactive oxygen species are another possible target, as ROS can signal neuroinflammation [238]. ROS has been successfully detected in rodents’ brains; ROS is currently being tested in transgenic models of human diseases such as AD [238]. There are numerous other suggested biomarkers for neuroinflammation, and continued research will be necessary to confirm their potential. These biomarkers are generally not expected to have true diagnostic potential on their own, as neuroinflammation is not specific to AD. The best strategy going forward will be using multiple of these biomarkers and pairing them with biomarkers specific to AD. Currently, the core believed biomarkers for AD include total tau, phosphorylated tau, and Aβ [239]. The analysis of these biomarkers together would then provide a better picture when it comes to the disease severity and progression.

## 5. Drug Development Targeting Neuroinflammation for Alzheimer’s Disease

Many studies have indicated that anti-inflammation drugs such as nonsteroidal anti-inflammatory drugs (NSAIDs) are associated with a lower risk of AD development [240,241,242]. Anti-inflammation has become a potential therapeutic target for AD drug development. According to the 2021 Alzheimer’s clinical trials report (https://www.alzdiscovery.org/uploads/media/ADDF-CTR-2021-06-singles.pdf) (accessed on 1 August 2022), 14.4% of the current clinical trials for AD are studying drugs to reduce chronic neuroinflammation. Those anti-inflammation drugs target several pathways to relieve neuroinflammation and other AD-associated pathologies, showing significant therapeutic potential for ameliorating and treating AD [243]. Table 2 summarizes currently available clinical trials that target neuroinflammation in patients with AD and MCI.

### 5.1. Immunotherapy

#### 5.1.1. Passive Immunotherapy

Genome-wide association studies (GWAS) have made a considerable contribution to the identification of AD genes. They have shown that more than 30 susceptible gene variants could increase the risk of developing late-onset AD [244]. Most of these genes are selectively or highly expressed on microglia, which include TREM2, ATP-binding cassette sub-family A, membrane 7 (ABCA7), complement receptor (CR1), and CD33. Several variants in TREM2, especially R47H, have been significantly associated with AD risk [245]. It has been indicated that TREM2 variants could induce the partial function impairment of the TREM2 proteins and further alter the microglial degradation of Aβ. Wang and colleagues (2020) have indicated that treating Aβ accumulation in AD mouse models expressing either the common variant or the R47H variant of TREM2 with TREM2 agonistic mAb, AL002, reduced Aβ plaques, and neuronal loss and improved cognitive function [246]. CD33 is also expressed in microglial cells, which can impair the microglia-mediated Aβ clearance, thus increasing the burden of Aβ plaques in the brain [247]. AL003, an anti-CD33 antibody that counteracts the CD33, has entered Phase I clinical trials and has now been completed. AL003 was generally found to be safe and well tolerated by healthy volunteers in a Phase I study, and a proof-of-concept Phase II study was considered for further investigation [248]. A recent study by Griciuc and colleagues (2019) to link TREM2 and CD33 has indicated that TREM2 downregulates CD33 that modulate microglial function in AD, thus further supporting targeting these receptors to treat neuroinflammation in AD [249].

#### 5.1.2. Active Immunotherapy or Vaccination

Another therapeutic strategy to treat AD is to actively stimulate the immune response. GV1001 is a peptide consisting of 16 amino acids derived from a fragment of the human telomerase reverse transcriptase (hTERT) sequence [250]. GV1001 was initially developed as an anti-cancer vaccine. Surprisingly, GV1001 has demonstrated its anti-inflammatory and antioxidant activity in non-cancer cells [250,251,252]. A series of studies have revealed the neuroprotective effect of GV1001 by blocking the Aβ oligomer-induced neurotoxicity and increasing the cell viability following exposure to oxidative stress in rat neuronal stem cells [250,251]. In addition, GV1001 reduces neuroinflammation by downregulating the production of pro-inflammatory cytokines by suppressing the activation of p38 MAPK and the suppression of NF-κB in vitro [252]. The completed Phase II clinical trial of GV1001 has demonstrated the treatment’s safety and potential beneficial effect in patients with moderate-to-severe AD [253]. However, more investigations with a larger clinical trial group are necessary [253]. Another Phase II clinical trial of GV1001 has been approved but not yet recruited (Clinical trial ID, NCT05189210).

Similar to GV1001, another anti-cancer vaccine, Bacillus Calmette-Guérin (BCG), has also been suggested to decrease the AD risk by enhancing the innate immune response and downregulating the systemic inflammation at the same time [254,255]. However, it remains unclear by which mechanism BCG reduced neuroinflammation, and further studies are still required. A Phase II study to evaluate the effects of BCG in adults with MCI and mild-to-moderate AD is currently recruiting (Clinical trial ID, NCT05004688).

### 5.2. Small Molecule Compounds

Numerous studies have proven the capability of small molecule compounds to modulate microglial phagocytosis, enhance Aβ degradation, and inhibit neuroinflammation, which attracts considerable attention in AD therapeutic development [256,257,258,259,260]. Several compounds have entered the clinical stage for further safety and efficacy examination. Neflamapimod (VX-745) is a highly selective inhibitor of the intra-cellular enzyme p38 MAPKα [261]. p38 MAPKα plays an essential role in regulating the production of TNFα and IL-1β, and other pro-inflammatory cytokines in the CNS [262]. p38 MAPKα modulates neuroinflammation by acting on both microglia and astrocytes in the brain [263,264,265,266,267,268]. In stress or disease status, the expression of p38 MAPKα on neurons induces Aβ-toxicity, synaptic dysfunction, tau, and neuroinflammation [256,257,258,259,260]. Neflamapimod, a high-specific antagonist of p38 MAPKα, has a substantial therapeutic potential to be developed as an anti-inflammatory drug for AD.

JNJ-40346527 is another small molecule compound targeting neuroinflammation to start Phase I clinical trials on MCI-AD patients. Mancuso et al. 2019 have indicated that JNJ-40346527 enters the brain and successfully inhibits microglia proliferation by selectively inhibiting the colony-stimulating factor-1 receptor (CSF-1R) in a mouse model. JNJ-40346527 reduced the production of pro-inflammatory cytokines, including TNFα and IL-1β [269]. In a tau-induced mouse model, JNJ-40346527 reduced the phosphorylation of tau, attenuated neuroinflammation, improved the spinal motor neurons, and recovered the gene expression levels on microglia after chronic treatment [269]. Additional therapeutic drugs that are currently in development and are in clinical trials are listed in Table 2.

### 5.3. Other Pharmacological Treatments of AD

Due to the complicated pathology and the multiple targets of AD, combination therapy targeting diverse factors concurrently looks more promising than individual therapy. ALZT-OP1, a combination therapy drug, has completed the Phase II clinical trial assessing the effects on AD patients and cognitively normal healthy subjects (Table 2). ALZT-OP1 is a combination of two FDA-approved drugs, cromolyn (ALZT OP1a) and ibuprofen (ALZT OP1b). Cromolyn is a small molecule mast cell stabilizer approved for treating asthma via an oral dry powder inhaler. Ibuprofen, an NSAID, significantly reduced the inflammation biomarkers and Aβ level in AD mouse models [270]. Another study indicated that ibuprofen could attenuate oxidative stress and enhance Aβ plaque clearance in the brain by inhibiting the COX enzyme to modulate the signaling cascades and thus activate microglial NADPH oxidase [271]. Cromolyn alone or its combination with ibuprofen (ALZT-OP1) reduced the levels of Aβ deposition by promoting microglia recruitment and phagocytosis [272,273]. Long insoluble Aβ species, Aβ_40_ and Aβ_42_, were almost abolished, while the shorter Aβ_38_ was increased in an APPSwedish-expressing Tg2576 AD mouse model [274]. In vitro cell assay has indicated the critical role of cromolyn in upregulating the microglial uptake of Aβ species [272]. These studies showed the neuroprotective role of ALZT-OP1 combination therapy in promoting the equilibrium of soluble and insoluble Aβ in AD pathology and its capability of ALZT-OP1 as an anti-inflammatory and anti-Aβ aggregation agent. In addition, these findings affirm that ALZT-OP1 reduced neuroinflammation by modulating the activation state of the microglial to enhance Aβ phagocytosis rather than producing pro-inflammatory cytokines, making it a promising potential AD therapeutic in both early and later-stage phases of the disease.

Findings from a preclinical study from our laboratory also demonstrated that another NSAID, etodolac, can be applied in combination therapy with α-tocopherol, an antioxidant, to reach an additive or synergistic therapeutic effect to prevent, slow, or treat AD [275]. According to the in vitro and in vivo data in the 5xFAD mice model, the combination therapy reduced the BBB leakage, decreased Aβ burden by modulating the APP processing towards neuroprotective and non-amyloidogenic sAPPα, and enhanced the expression of synaptic markers expression [275]. In addition, the combination decreased oxidative stress and neuroinflammation as determined by the synergistic reduction in astrogliosis, COX-2, and PGE2 [275]. These studies provided compelling evidence of combination therapy as a potential strategy for treating AD.

### 5.4. Non-Pharmacological Treatments of AD

Beyond therapeutic drugs, mounting scientific evidence has indicated that long-term exposure to the Mediterranean diet (MedD) is associated with lower risks of dementia [276]. One of the major characteristics of MedD is the daily consumption of extra-virgin olive oil (EVOO), which has been acknowledged for its anti-inflammatory and anti-oxidative capabilities [277,278,279,280,281,282]. Preclinical studies have shown that daily consumption of EVOO protects early-stage AD mice from Aβ-related pathology development by restoring their BBB function through neuroinflammation reduction and autophagy induction [283,284,285]. Inhibition of NLRP3 inflammasome activation and RAGE/HMGB1 by EVOO resulted in reduced neuroinflammation and Aβ deposition as downstream events [99,285]. Chronic exposure to EVOO as a dietary supplement was expected to stop or slow AD progression. Clinical studies have indicated that long-term intervention with EVOO significantly improved cognitive function [286,287]. The plasma serum samples of MCI patients that consumed EVOO for 12 months significantly reduced IL-6 and TNF-α levels compared with MCI patients in the control group, demonstrating the potential of EVOO acting as an anti-inflammatory agent [287]. This comprehensive evidence further proved the potential of EVOO to prevent dementia and slow the progression of MCI to AD.

Antioxidants such as flavonoid supplements can also decrease neuroinflammation in AD, yet their use is limited due to low bioavailability [288]. A similar protective effect was proposed with curcumin supplements in another study. It decreased neuroinflammation by reducing lipopolysaccharide-induced neuroinflammation in male Sprague–Dawley rats treated intraperitoneally with 300 mg/kg/day curcumin for 14 days. The reduction in oxidative stress modulated the JNK/NF-κB/Akt signaling pathway to reduce neuroinflammation and alleviate cognitive impairment [289]. Vitamin C reduced neuroinflammation in colchicine-induced AD rats in lower doses (200 and 400 mg/kg BW) but not at higher doses (600 mg/kg BW) because of decreased ROS. This reduction was evident by the reduced inflammatory biomarkers TNF α, IL 1β, ROS, and nitrite in the brain hippocampus and serum [290]. Furthermore, omega-3 polyunsaturated fatty acids were linked to a lower risk of neuroinflammation by reducing IL-1, TNF-α, IL-6, iNOS, and COX-2 [291]. Moreover, and as mentioned above, microbiota could positively affect neuroinflammation; for example, treatment with *Clostridium butyricum* for four weeks reduced neuroinflammation in APPswe/PS1dE9 (APP/PS1) transgenic mice by reducing microglial activation and cytokines release such as IL-1β and TNF-α in mice brain [292]. Yet, while dietary supplements and probiotics might positively affect neuroinflammation, additional studies for characterization and mechanisms elucidation are required to provide therapeutically applicable guidelines.

Physical exercise is another lifestyle modification that could target neuroinflammation in AD [293]. Aerobic exercises reduced neuroinflammation in rats’ brains after induction with lipopolysaccharide and showed a reduction in apoptosis by reducing expressions of Bax and Bcl-2 and caspase-3 [294]. Another study has demonstrated that physical exercise can improve the cognitive function of rats with chronic cerebral hypoperfusion (CCH) by promoting the neuroprotective role of astrocytes by alleviating the phosphorylation of ERK and JNK proteins induced by CCH [295]. In addition, resistance exercise reduced neuroinflammation in 3xTg mice by reducing microglial activation and the inflammatory markers IL-1β and TNF-α [296]. Moreover, studies using treadmill exercise demonstrated that exercise suppressed microglial activation and neuroinflammation by activating the TREM2 pathway leading to improved memory in AD-induced rats [297]. Collectively, these findings support that physical exercise could help maintain the regulatory mechanisms of brain homeostasis through anti-inflammatory mechanisms.

## 6. Conclusions

In this work, we reviewed updates on the neuroinflammatory role of glial cells in AD. Neuroinflammation contributes to the acute-phase response stage of AD, where the brain prevents infection and attempts to return to homeostasis; however, it acts detrimentally in later stages. The chronic stimulation of microglia and astrocytes activates an immune response which could further trigger an increase in Aβ deposits, tau hyper-phosphorylation, and cerebral amyloid angiopathy (CAA). We also explored different mechanisms of neuroinflammation, diagnostic tools, and potential therapeutic interventions in preclinical stages and those currently in clinical trials. We anticipate that the improved harness of neuroinflammation can lead to developing novel therapies and medications for those suffering from AD.

Currently, there is no treatment for AD, and current medications only provide symptomatic relief, while for aducanumab, additional evidence is needed on its effectiveness. Thus, the need continues to develop treatments that target the neuroinflammation process and related mechanisms associated with AD. While the prevention of AD is considered the best approach to fight AD, early specific diagnostic tools to identify patients who are at high risk are necessary.

In conclusion, neuroinflammation, among other pathological hallmarks, plays an important role in AD progression that should be researched for its specific contribution to the disease, its utilization as a diagnostic tool for AD progression, and as a therapeutic target to cure the disease. While the cause of AD is yet to be clarified, ongoing research continues to demonstrate the critical role of glial cells in the initiation and progression of AD, which upon chronic activation, causes additional damage to the neurovascular unit and thus contributes further to the neurodegenerative process in AD. Therefore, understanding the role of microglial and astrocytes in the pathology of AD and their response to treatment could help restore their function to support the neurons and the BBB.

## Figures and Tables

**Table 2:** 2021 Clinical Trials of anti-inflammatory drugs in AD and MCI patients.

Drugs	Clinical Stage	Status	Mechanism	Clinical Trial ID
AL003	Phase I	Completed	Anti-CD33 antibody	NCT03822208
JNJ-40346527	Phase I	Not yet recruiting	Selective inhibitor of the CSF-1R tyrosine kinase	NCT04121208
Salsalate	Phase I	Active, not recruiting	NSAID	NCT03277573
Sirolimus	Phase I	Recruiting	mTOR inhibitor	NCT04629495
XPro1595	Phase I	Completed	Neutralize sTNF	NCT03943264
AL002	Phase II	Recruiting	Anti-TREM2 antibody	NCT04592874
ALZT-OP1(cromolyn and ibuprofen)	Phase II	Completed	Combination of a cytokine release suppressor and an NSAID	NCT04570644
Bacillus Calmette-Guerin (BCG)	Phase II	Recruiting	Systematic immune activation	NCT05004688
Daratumumab	Phase II	Recruiting	Anti-CD38 antibody	NCT04070378
GV1001	Phase II	Not yet recruiting	Systematic immune activation	NCT05189210
Lenalidomide	Phase II	Recruiting	Modulate innate and adaptive immune responses; inhibit BACE1	NCT04032626
Montelukast	Phase II	Active, not recruiting	Inhibit the CysLT1 receptor	NCT03991988
Tacrolimus	Phase II	Withdrawn (COVID restrictions)	Calcineurin Inhibition	NCT04263519
VX-745	Phase II	Completed	Selective p38 MAPKα inhibitor	NCT03402659
NE3107	Phase III	Recruiting	ERK inhibitor	NCT04669028

## Data Availability

Not applicable.
